# Effectiveness of Facebook-Delivered Lifestyle Counselling and Physical Activity Self-Monitoring on Physical Activity and Body Mass Index in Overweight and Obese Adolescents: A Randomized Controlled Trial

**DOI:** 10.1155/2015/159205

**Published:** 2015-12-01

**Authors:** Heidi Ruotsalainen, Helvi Kyngäs, Tuija Tammelin, Hanna Heikkinen, Maria Kääriäinen

**Affiliations:** ^1^Research Unit of Nursing Science and Health Management, University of Oulu, Oulu, Finland; ^2^Medical Research Center Oulu, Oulu, Finland; ^3^University Hospital of Oulu, Oulu, Finland; ^4^LIKES-Research Center for Sport and Health Sciences, Jyväskylä, Finland; ^5^Department of Mathematical Sciences, University of Oulu, Finland

## Abstract

*Background*. The aim was to evaluate the effects of a 12-week, Facebook-delivered lifestyle counselling intervention, with or without physical activity self-monitoring, on physical activity and body mass index (BMI) in overweight and obese 13–16-year-old adolescents.* Methods*. Three-arm randomized controlled trial. Participants (*n* = 46) were randomly assigned to intervention and control groups: one group received Facebook-delivered lifestyle counselling and monitoring of their physical activity (Fb + Act, *n* = 15), whereas a second experimental group received the same Facebook-delivered lifestyle counselling without self-monitoring (Fb, *n* = 16) and a third group served as the control group (*n* = 15). Objective and self-reported physical activity assessment were used. Nonparametric statistical tests were used.* Results*. There were no significant intervention effects in terms of changes in physical activity levels or BMI from baseline to the 12-week postintervention measurements between the intervention and control groups. The Fb + Act group had lower sedentary time on weekdays compared to the control group during postintervention measurements (*p* = 0.021), but there was no interaction between time and group.* Conclusions*. Interventions were not effective at increasing physical activity in overweight and obese adolescents. Before implementing such interventions, more evaluations on their effectiveness are needed. This trial is registered with ClinicalTrials.gov identifier NCT02295761 (2014-11-17).

## 1. Introduction

The percentage of overweight and obese children and adolescents has increased [1] and it is globally recognized as a serious health problem [2]. Overweight and obese adolescents are at risk of getting chronic illnesses [3], and their mental health and self-esteem are affected as well [4]. During adolescence, the frequency of participation in physical activity and sports declines remarkably and the amount of sedentary time increases [5, 6]. Less than 20% of adolescents meet physical activity guidelines [6, 7], which suggest that they should engage in at least 60 minutes of moderate to vigorous physical activity (MVPA) daily [8]. Physical activity in adolescence has many health benefits [9] and it is indicative of higher levels of adult physical activity [5]. In adulthood, physical inactivity or sedentary behaviour is associated with higher mortality rates and several common diseases, such as coronary heart disease, diabetes, certain types of cancers, and mental health problems [10]. Sedentary behaviours (waking activities with an energy expenditure of ≤1.5 metabolic equivalents) [11], such as too much sitting or time spent in front of the TV and computer screens, are one reason for obesity [12]. Sedentary behaviour and its negative impact on health have been recognized in health promotion campaigns [13]. Prolonged sedentary time is independently associated with negative health outcomes [14]. It is important that health promotion among overweight and obese adolescents includes both physical activity promotion and reducing sedentary time and not just weight control.

Schools can be effective environments for health [15] and physical activity promotion [16]. Lifestyle counselling for adolescents and their parents about healthy lifestyles is provided by school health care services [17, 18]. Lifestyle counselling interventions for obese and overweight adolescents include multiple components, such as providing (1) information about physical activity, sedentary behaviour, and nutrition, (2) social support, and (3) tools for enhancing behavioural management skills [19]. Sources of social support include support from peers, friends, school nurses or health care experts, and parents [20, 21]. Parent participation increases the effectiveness of school-based counselling with respect to adolescent physical activity [22, 23]. Behavioural management skills include reinforcing behaviours through self-rewarding mechanisms [24], goal setting [25–27], and problem solving [27, 28], as well as self-monitoring physical activity [29], to increase physical activity levels and reduce sedentary behaviour and BMI.

However, school health care services are not providing effective lifestyle counselling due to insufficient time and a lack of evidence-based knowledge and counselling skills [30]. Adolescents' lack of motivation and willingess to change their lifestyles are common barriers [31]. Novel interventions for promoting physical activity among adolescents are needed for school health care services. Online social networks are feasible platforms for intervening with adolescents [32]. Online social networks that promote health are widely and publicly available [33]. Conducting interventions via the Internet has a high potential for success because use of the Internet between the years 2000 and 2014 has increased by more than 400% in Europe. In Finland, more than 43% of the population use Facebook [34]. Adolescents spend a great deal of time engaging in sedentary behaviour [35], with social media in particular [32]. Adolescents use different kinds of social media applications on a daily basis. Hence, lifestyle counselling could be made more readily available for them via social media. Providing counselling via social media could also free up valuable time for the school nurse to address other matters. Social media-based interventions can provide an alternative means for promoting health care, especially in rural areas [36], like the northern part of Finland, where distances are great. While computer- and web-based interventions can promote physical activity among adolescents, according to a review by Hamel et al. [37], websites should be of a higher quality [33]. Freely accessible physical activity intervention websites should include the opportunity for self-monitoring, goal setting, and providing feedback. Social media like Facebook, Twitter, YouTube, and smartphone applications are widely used on physical activity intervention websites, even though many social media have yet to be tested [33]. Besides social media, technical devices can motivate adolescents to increase their daily physical activity. Pedometers increase physical activity and decrease BMI among adults [38], and pedometers have been used successfully with adolescents in a variety of ways to promote activity [39]. With overweight or obese adolescents, however, the effectiveness of promoting physical activity in these ways remains unclear [19].

In the present study, we developed a 12-week Facebook-delivered lifestyle counselling intervention to promote physical activity and reduce sedentary time and BMI among overweight and obese adolescents. As far as we can tell, this is the first such study to assess the effects of such an intervention on objectively measured levels of physical activity. This study evaluates the effects of a Facebook-delivered lifestyle counselling intervention, with or without the monitoring of physical activity, on physical activity among overweight and obese adolescents (sedentary time or very light physical activity, as well as light, moderate, and vigorous physical activity) and BMI. We hypothesized that (1) the Facebook-delivered lifestyle counselling intervention and self-monitoring of physical activity (Fb + Act) would be more effective at improving physical activity and reducing sedentary time and BMI compared to a Facebook-delivered lifestyle counselling intervention without physical activity self-monitoring or a control group; and (2) the Facebook-delivered lifestyle counselling intervention would be more effective at improving physical activity and decreasing sedentary time and BMI compared to a control group.

## 2. Materials and Methods

We used a three-arm randomized controlled trial design with two intervention groups and a control group. One of the intervention groups received Facebook-delivered lifestyle counselling and the Polar Active physical activity monitor (Fb + Act) to help participants self-monitor their daily physical activity, while another experimental group received Facebook-delivered lifestyle counselling without physical activity monitoring (Fb) and the third group served as a control group (CG). This study has been registered at ClinicalTrials.gov. The local ethics committee of the University Hospital of Oulu approved the study protocol. Adolescents and their parents were familiarized with the study and had a chance to present questions or thoughts to the researchers. Those who were willing to take part in the study received a consent form to sign. Written informed consent was obtained from the study participants before the baseline examinations. For adolescents under 15 years of age, written informed consent was obtained from their parents.

### 2.1. Intervention

#### 2.1.1. The Facebook-Delivered Lifestyle Counselling Intervention

The Facebook-delivered lifestyle counselling intervention for overweight and obese 13–16-year-old adolescents was developed using the Medical Research Council's [40] development-evaluation-implementation process framework [41, 42]. The Facebook-delivered counselling intervention was evaluated for feasibility via an expert panel, which consisted of health science experts (*n* = 4), 15–17-year-old assistant nursing students (*n* = 5), and one 13-year-old adolescent. The intervention was targeted primarily at promoting physical activity among adolescents and reducing their sedentary time and secondarily at reducing their BMI. The theoretical basis for the counselling intervention was based on (1) the theory of compliance [20], (2) a systematic review of earlier physical activity interventions [19], and (3) interviews. According to the theory of compliance, an adolescent's role in this intervention is as an active, intentional, and responsible participant who works to maintain his/her healthy lifestyle in collaboration with health care experts [20]. A systematic review of earlier interventions showed that counselling could be effective at increasing physical activity [19]. We developed the components of this intervention based on the review. We modelled the informational themes of the lifestyle counselling intervention and delivery method by interviewing obese adolescents, their parents, and nursing staff about the barriers to and opportunities for being physically active.

The intervention period was during the school term in the spring of 2012 and the duration was 12 weeks. The intervention was delivered via Facebook. Two closed Facebook groups were established: one for the adolescents and one for their parents. A physiotherapist acted as the tutor every weekday and a dietitian visited the group once a week. The tutor shared material and read posts, commented on them, and answered questions. The Facebook-delivered lifestyle counselling intervention included the following components: (1) informational support, (2) social support, (3) behavioural management skills, and (4) menu and tailored exercise programme suggestions. The informational support included six themes pertaining to physical activity and diet. While physical activity played a major role in the intervention, we also included dietary tips and advice to supply the content. The informational support themes were published on Facebook © fan pages (one for adolescents and a separate one for parents) once every two weeks, and it was shared with parents and adolescents at the same time. Those particular pages were public, but the participant groups were closed. The informational support themes were as follows: (1) general information about physical activity and dietary recommendations, (2) sedentary behaviour and changes in adolescents' cultural environment, (3) social support, (4) adolescents' living environment and their ability to be physically active, (5) adolescents' functional ability, and (6) their ability to maintain a physically active lifestyle. Social support included emotional and material support and social support from the tutor, dietitian, parents, peers, and friends.

Public and private discussions with parents and adolescents were held weekly. They dealt with how to motivate adolescents to change their habits regarding a physically active lifestyle and reduce their daily sedentary time. Parents were encouraged to give emotional and material support to adolescents to change their physical activity patterns. Participants were encouraged to discuss the related issues with each other in the social media environment and discussion channels. Behavioural management skills included problem solving related to the barriers to being physically active; motivating questions and ideas were shared via posts. Tools for posting comments, using questionnaires, and sharing materials (news, videos) were used in the adolescent and parent Facebook groups. Adolescents and their parents also had the opportunity to make tailored exercise programme suggestions ranging from expressing the need for a physiotherapist to making menu suggestions from dieticians via Facebook. The tailoring adopted a client-centred approach based on discussions with adolescents and their parents.

#### 2.1.2. Facebook-Delivered Lifestyle Counselling with Physical Activity Self-Monitoring

Another experimental (Fb + Act, *n* = 15) group received the same Facebook-delivered lifestyle counselling and received the Polar Active physical activity monitor to self-monitor their daily physical activity. The Fb + Act group used the monitor on their wrists for 12 weeks. The monitor continuously measured their daily activity at different activity levels (sedentary or very light, light, moderate, vigorous, and vigorous plus). Instructions on how to use monitor and measure their daily physical activity levels were given to adolescents [43]. Adolescents could monitor their daily physical activity with the device, which had a screen that displayed daily minutes of physical activity (at least moderate intensity) and a bar showing the amount of activity in relation to a recommended level. The activity monitor tracked the intensity and duration of their daily physical activity as well as their sedentary time in minutes per day (sitting, walking with very low intensity). The monitor showed the user's individual daily steps and total daily energy consumption in calories (Kcal). The Polar Active physical activity monitor has been used to activate children [44] and scholars have found the device to be quite beneficial [45].

#### 2.1.3. Control Conditions

The control group (*n* = 15) participated in measurements at the baseline and after intervention at postintervention measurements. We provided them with feedback on their physical activity after postintervention measurements. The control group as well as the intervention groups received counselling from the school nurse regarding their health problems, if they so required it. All groups were treated in a similar way throughout the study.

### 2.2. Sampling Design

**Figure 1:**
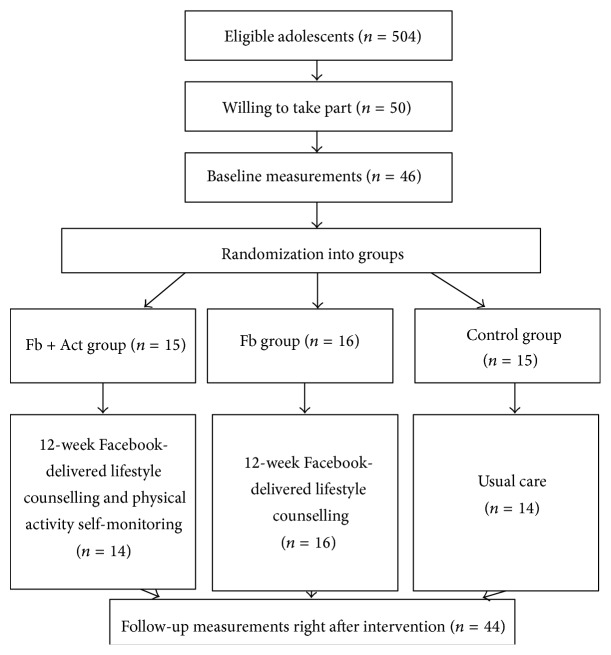
Flow of participants through the trial.

The sample was collected from health care services for a school district in northern Finland and participants were recruited from nine schools. There were three groups (Fb + Act group, Fb group, and the control group) with data collected at two different time periods: at the baseline and during postintervention measurements 12 weeks later. The eligibility criteria for the participants were that they must be between 13 and 16 years of age, be overweight or obese (weight-for-height 20% beyond the mean, Finnish national overweight and obesity cut-off point for 2010), and not have any diagnosed mental health problems or any other health issues and that they would be willing to take part in the study. School nurses (*n* = 9) screened all 13–16-year-old students (*N* = 2270), and invitation letters to participate in the study were sent to those adolescents (*n* = 504) and their parents who were eligible based on the inclusion criteria. To obtain a larger number of participants, a second invitation was sent out. Altogether, 50 adolescents and their parents participated in an information event, which was held at the local schools in January of 2012. Forty-six of them ultimately were willing to take part in the study and enrolled for the baseline examinations. Two adolescents and their parents from the control group and from the Fb + Act group dropped out after the baseline measurements (Figure 1). Adolescents were motivated to complete the study when it was promised that 36 Polar Active physical activity monitors would be awarded via a raffle after the study.

#### 2.2.1. Data Collection Procedures

Data collection was performed in February 2012 (baseline) and May-June 2012 (postintervention measurements). The researcher's assistant collected the baseline data using questionnaires and during face-to-face weight and height measurements as a part of information events held at the participants' schools. The researcher gave the Polar Activity monitors to the participants for 21 days to objectively measure their daily physical activity. The physical activity monitor was blinded during the measurement period, so it essentially worked as a watch and the subjects were not able to self-monitor their physical activity during the measurement period. After the baseline measurement, blocks were formed using nuisance factors of age, gender, and self-evaluated physical activity (days out of the past seven with at least 60 minutes MVPA). The researcher assigned the participants to different groups. Within each block, participants were randomized into the intervention groups (Fb + Act, Fb) or the control group (1 : 1 : 1). Sealed envelopes were sent to the participants with the information about which group they belonged to after the baseline measurements. Blinding the subjects was not possible for this kind of behavioural study. Data collection was performed at the postintervention measurements in the same way as it was at the baseline assessment.

### 2.3. Measures

#### 2.3.1. Objectively Measured Physical Activity

Physical activity was assessed using the Polar Active physical activity monitor (Polar Electro Kempele Oy, Kempele, Finland). The Polar Active physical activity monitor is a uniaxial accelerometer worn on the wrist that determines the physical activity of children and adolescents [46]. The device integrates the total amount of acceleration in one dimension while also measuring the duration and intensity of physical activity. The intensity of physical activity is defined by the metabolic equivalent of tasks (MET), which are divided into five levels of physical activity: sedentary time or very light physical activity (< to 2 MET), light physical activity (2–3.49 MET), moderate physical activity (3.5–4.99 MET), vigorous physical activity (5–8 MET), and vigorous plus physical activity (>8 MET). The Polar Activity monitor records physical activity continuously in 30-second bouts. Activity measurements were taken for 21 days, and the adolescents were asked to wear the activity monitor for 21 consecutive days and nights, even during water-based activities. The wearing time shows the valid days in this study, and one valid day required at least 500 minutes/day. The data from the activity monitor were downloaded to a computer program (Polar GoFit), where it was then transferred to a statistics program (excel). The data were transformed so that every participant was given their own average values (minutes) according to the different physical activity level during weekdays and weekends and in total. The wearing time was calculated by computing all the physical activity levels together. The wearing time does not include the participants' sleeping time. An objectively measured MVPA was calculated by combining moderate, vigorous, and vigorous plus physical activity. Objectively measured sedentary time or a very light percentage (%) of physical activity of wearing time was calculated based on the daily levels of very light physical activity in relation to the daily wearing time for the daily physical activity monitor because the wearing time is related to very light levels of physical activity.

#### 2.3.2. Self-Reported Physical Activity and Screen Time Questionnaire

We used a physical activity questionnaire to assess adolescents' self-reports of their physical activity and screen time. The questionnaire included demographics. The adolescents also self-evaluated their physical activity and screen time by answering questions used earlier in the WHO Health Behaviors in School-Aged Children study [7]. Self-reported moderate-to-vigorous physical activity (MVPA) was measured via the following question: “Over the past 7 days, on how many days were you physically active for a total of at least 60 minutes per day?” The response alternatives varied from 0 to 7 days. The question included a description of what kinds of physical activity should be taken into account and examples of MVPA were also given. The test-retest agreement for self-reported MVPA has been very good (ICC = 0.82) [46]. Self-reported screen time was measured via the following question: “How many hours a day do you usually (a) watch television (including DVDs and videos), (b) play computer or video/console games, and (c) use a computer (for purposes other than playing games, e.g., e-mailing, chatting or surfing the Internet or doing homework)?” There were separate response options for weekdays and weekend days and the response alternatives varied from 0 to 5 hours or more. The test-retest agreement for watching television (ICC = 0.72–0.74) and for playing computer or video/console games (ICC = 0.54–0.69) was significant, while it was fair to moderate for using a computer (ICC = 0.33–0.50) [46]. Self-reported daily screen time during weekdays and on weekend days was calculated by adding these three questions together.

*Body Mass Index*. Height was measured using a wall-mounted stadiometer and weight was measured with a floor scale. The body mass index was calculated according to the following formula: BMI = weight (kg)/(height (m))^2^. The baseline age was expressed as a decimal and calculated from the reported birthdate to the date when the baseline survey was completed.

### 2.4. Analysis

All variables were evaluated for normality using the Kolmogorov-Smirnov test, and the variables were nonparametric. Changes in objectively measured and self-reported physical activity and BMI from baseline to the 12-week postintervention measurements between groups were analysed using Kruskal-Wallis test [47]. Differences between the groups in terms of objectively measured and self-reported physical activity, BMI, gender, and age at baseline and during the postintervention measurements were tested using the Kruskal-Wallis test for continuous variables and Fischer's exact test for categorical variables. If a statistical significance was found between the groups during the postintervention measurements, we performed a Mann-Whitney *U* test to assess the pair of groups for which the difference was significant and then carried out a Bonferroni correction for the *p* value [48]. All analyses were performed using IBM SPSS Statistics version 20.0 (SPSS, Chicago, IL), with the significance level set at *ρ* < 0.05.

Two subjects did not wear the activity monitor long enough to reach a valid time during the baseline measurements and one subject did not wear it long enough for the postintervention measurements. In this case, the missing data were replaced using the means from all participants' physical activity data from the postintervention measurements [49]. For those who withdrew (*n* = 2), we analysed the data only at the baseline and included them in the results for the baseline; therefore, they were not considered when reporting the changes.

## 3. Results

### 3.1. Participant Profile

Table 1 presents a summary of the participants' background characteristics at the baseline assessment. There was no difference in objectively measured physical activity or in self-reported MVPA, screen time, or BMI between the study groups. The mean age of the adolescents was 14.7 years (SD 0.8), with a mean BMI of 28.1 (SD 5.7). The majority of the participants were girls (70%). Most of the adolescents lived with both of their parents (conjugal family 70%), who in most cases had attended college or a vocational college (62% of mothers and 58% of fathers) or had received a bachelor's level degree from the university (22% of mothers and 20% of fathers). The adolescents wore the Polar Activity monitor for, on average, 13 weekdays and 5 weekend days at the baseline and for 12 weekdays and 5 weekend days at the posttest.

### 3.2. Effects of the Intervention

The changes in objectively measured and self-reported physical activity and BMI from baseline to the 12-week postintervention measurement are reported in Table 2. There were no significant differences between the intervention group and control group in terms of the changes in objectively measured (sedentary time or very light physical activity, light, moderate, and vigorous physical activity) or self-reported physical activity, screen time, or BMI from the baseline to the 12-week postintervention measurements.

**Figure 2:**
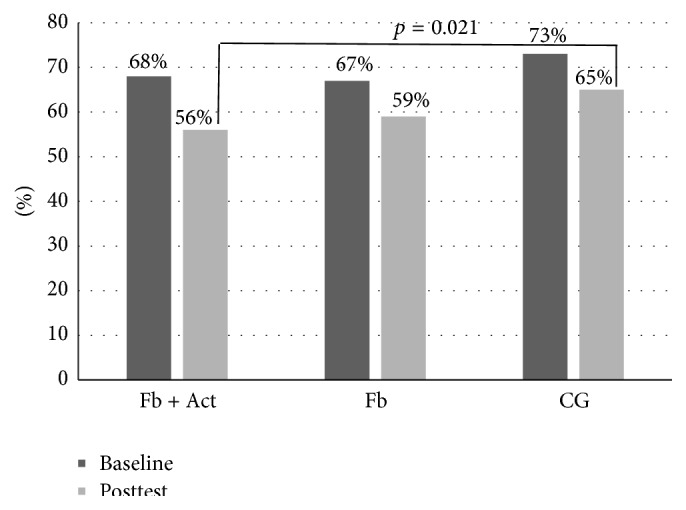
Daily time spent engaged in sedentary or very light physical activity on weekdays (%) at baseline and posttest.

Sedentary time or the percentage (%) of very light physical activity during wearing time on weekdays was statistically different between groups during the postintervention measurements (*p* = 0.025). The difference between the Fb + Act group and control group (*p* = 0.021, 95% CI: 0.72, 17.6) was significant at the postintervention measurements (Figure 2). The Fb + Act group had lower amounts of sedentary time or a lower percentage of very light physical activity during wearing time on weekdays. We did not find statistically significant differences between the groups during postintervention measurements for very light, light, moderate, vigorous, and vigorous + or moderate-to-vigorous physical activity. The mean values and standard deviations for physical activity and BMI measurements at baseline and after 12 weeks and the overall changes are reported in Table 2.

For self-reported physical activity, the number of days out of the past seven with at least 60 minutes of MVPA and screen time (total or divided into different modes or weekdays/weekends) did not differ between groups during the postintervention measurements and we did not observe any significant changes between the groups. Mean values and standard deviations for self-reported physical activity and screen time are reported in Table 3.

## 4. Discussion

The 12-week Facebook-delivered lifestyle counselling intervention with or without the self-monitoring of physical activity did not have an effect on the objectively measured or self-reported physical activity or BMI among the overweight or obese adolescents. However, the Facebook-delivered lifestyle counselling intervention with self-monitoring of physical activity decreased the time that adolescents spent engaged in sedentary or very light physical activity on weekdays by more than 11%, but compared to the control or Fb groups the change was not significant. Reducing sedentary behaviour and daily sedentary time may positively influence adolescent health. Studies have demonstrated associations between sedentary behaviour and health outcomes, such as cardiovascular diseases, an adverse metabolic profile, and obesity [50, 51]. However, the associations between sedentary behaviour and health outcomes are complex [14]. Lifestyle counselling for overweight and obese adolescents should not only recommend that adolescents be sufficiently physically active, but also discuss overall daily physical activity and adhering to healthy lifestyles. The Facebook-delivered lifestyle counselling intervention included information for adolescents and their parents about sedentary behaviour and changes in modern adolescent's cultural contexts, such as screen time, too much sitting, gaming, and communications with friends only via social media. The tutor persuaded adolescents to change their sedentary behaviour through problem solving and searching for opportunities to be physically active and challenged them to reduce their daily sedentary time. The physical activity monitor could motivate overweight and obese adolescents to track their daily physical activity and sedentary time and make changes in their daily life if what they currently do is not sufficient. However the evidence that the monitor actually helps reduce sedentary time needs to be further studied [13].

Increasing physical activity among overweight or obese adolescents seems to be complex. Physical activity counselling and exercise interventions with overweight and obese adolescents have only impacted their levels of physical activity to a minor extent [19]. Likewise, health behaviour interventions delivered via the Internet have only had a limited impact on participants' level of physical activity [52]. In the current study, the total increase in MVPA in the Fb + Act group was 2.6 min/day, whereas for the Fb group it was 3.0 min/day and for the control group it was 1.3 minutes/day. An earlier meta-analysis observed on average approximately a four-minute increase in MVPA per day among adolescents [53], which is insufficient for reducing the BMI [54] and the effect on, for example, blood pressure is minimal [54]. In an earlier RCT study, the use of a pedometer with behavioural skill management counselling did not affect overweight adolescents' physical activity either [55]. Trials targeted exclusively at overweight or obese adolescents have been slightly more effective at increasing total physical activity than trials targeted at normal weight adolescents [53], and MVPA improvements have been about six minutes [56].

In this study, the lifestyle counselling intervention with or without the monitoring of physical activity did not affect adolescent's BMI. Minor changes were noticed within the groups. We observed a decrease in BMI in the Fb group among 12 out of the 16 participants who received the Facebook-delivered lifestyle counselling intervention. These results are encouraging and the Facebook-delivered lifestyle counselling intervention should be used to test a larger number of participants. It would also be beneficial to do a follow-up assessment to evaluate whether the BMI remains the same later. Weight reduction is not the first objective in obesity management among adolescents. Researchers recommend putting a stop to continuously increasing weight gain [57]. Interventions delivered via the Internet have positively affected obese adolescents' BMI [58, 59]. In Facebook-delivered counselling interventions, a tutor persuades adolescents to maintain healthy lifestyles at the end of the intervention period. The follow-up assessment is important for evaluating how long the positive changes will last and whether the adolescents need some supporting interventions to maintain their lifestyle change.

When developing interventions for obese and overweight adolescents, it is necessary to remember that obese and overweight adolescents may not consider physical activity important, or their attitudes towards physical activity may be more negative than in their normal weight counterparts [60]. Obese adolescents' knowledge about physical activity does not differ from that of normal weight adolescents [61]. However, their attitudes may be the reason why increasing physical activity among obese and overweight adolescents is challenging. There can also be unique barriers to being physically active with overweight adolescents [62], for example, their motor skills [63]. Therefore, we recommend that obese adolescents could start to become more physically active by first reducing their daily sedentary time instead of starting immediately with high-intensity physical activity. We found that adolescents participating in this study were sufficiently physically active already at baseline according to our baseline measurements (MVPA mean > 60 minutes/day). So perhaps the adolescents who participated could have been interested in exercising, sports, and maintaining a healthy lifestyle even at the baseline. More specific counselling should have been done to promote physical activity among the adolescents and we should have asked them about the usefulness of this kind of intervention. This would have helped us further develop our Facebook-delivered lifestyle counselling intervention. Additionally, information on the themes should be more specific and more tailored information should be given to the participants to help them change their physical activity patterns.

The adolescents in this study still spent most of their days (60%) engaged in sedentary or very light physical activities (>10 hours/day). Overweight and obese adolescents tend to be more sedentary than normal weight adolescents [64], and their attitudes towards physical activity are less positive [61]. Approximately 10% of obese and 16% of overweight adolescents are sufficiently physically active [64]. Compared to their normal weight counterparts, who were studied in another Finnish study with the same physical activity monitor [65], the adolescents in this study were less physically active. Whereas the average MVPA time for normal weight adolescents was 99 (SD 40) minutes per day [65], in this study the MVPA for all adolescents was 70 (SD 28) minutes at baseline.

When evaluating complex interventions in nursing, it would also be important to evaluate the effectiveness of the process [40], for example, participants' adherence in the programme. In this study, we did not measure the extent to which participants continued to use the activity monitor, so we do not know for sure whether the adolescents in the Fb + Act group used the activity monitor or not during the intervention period. In this study, we also did not evaluate participants' usage of the Internet for the purpose of counselling (posting, sharing, or visiting in group). However, 12 out of the 31 adolescents who received the lifestyle counselling intervention ordered physical exercise programmes. The tutor felt that some of the adolescents adhered to the programme and others did not.

The low participation rate in this study (9%) may partly explain why there was no significant difference between the experimental and control groups, even though we did notice small changes within the groups (e.g., BMI). In the recruitment process for intervention studies, it is important to know the correct size for a study population so that the study will have the necessary statistical power. Researchers should recruit committed personnel for the study [66]. Power and sample size calculation could be done to estimate the correct sample size for the study [67]. In this study, we decided to invite all adolescents who were overweight or obese (*n* = 504) because we knew that it would be difficult to get enough adolescents involved otherwise. However, we obtained a smaller than expected number of participants (*n* = 46). The study represents 23% of adolescents for that particular hospital district. Participation loss may also explain the fact that obese and overweight adolescents have less positive attitudes towards physical activity than normal weight adolescents [60]. The recruitment of adolescents for intervention studies is problematic [68]. Marketing and promoting this type of intervention should have been done more effectively. During the recruitment phase, cooperation with school nurses is important in order to gain contact with the adolescents [69]. In this study, the school nurses recruited adolescents for the study because of ethical reasons, but they did not receive any extra compensation for doing so. Perhaps, then, they were not sufficiently motivated to recruit participants. Also, conducting multicenter trials could be a solution in the future for obtaining sufficient numbers of participants from among the study population in this kind of study [68]. We sent out written information about the study and we tried to be as sensitive as possible by not using words like overweight or obese because of issues of sensitivity and stigmatization [70]. The adolescents' own motivation as well as their parents' motivation may also have affected whether or not they chose to sign up for this study.

Common problems in evaluating complex interventions are small participation rates and staffing-issues [71]. That could have been predicted by thoroughly piloting an intervention with the target group [40]. According to the MRC [40] it is possible with the intervention development process to take steps backwards from the evaluating phase to the developing phase. In this case, we could technically call this study a feasibility study, but we do not consider that to be ethically correct [72]. Because we did the feasibility assessment beforehand, the aim of this study was to evaluate effectiveness.

Ten experts from the field of health sciences and information technology, several nursing students, and one adolescent evaluated the intervention informational content themes and how to deliver them before the intervention began. We wanted to make sure that the informational content was readable, clear, and appropriate. After the expert review, we made a few changes to spelling and we shortened the text based on results from the expert panel. As it turned out, the Facebook-delivered lifestyle counselling intervention was easy to conduct, cheap, and easy to use. Thus, the intervention did not require expensive programmes. However, when evaluating its effectiveness, we faced challenges during the recruitment process, in collecting data and in motivating participants to take part in the discussions and share posts. We faced such challenges even though a feasibility assessment had been done. The theoretical basis of the intervention was systematic review [19] and the theory of compliance [20], and the informational content themes were based on interviews with obese adolescents.

When evaluating the effectiveness of the intervention with lager numbers of subjects, these aspects should be taken into account. This intervention needs to be developed further. Its effectiveness, process, and overall cost-effectiveness should be evaluated before implementing it as part of school health care services.

## 5. Conclusions and Future Directions

The findings reported in this paper suggest that a Facebook-delivered lifestyle counselling intervention targeted primarily at promoting physical activity among adolescents was not effective in increasing physical activity or decreasing BMI for those participating in the study. For future interventions, we suggest that lifestyle counselling for overweight and obese adolescents should not only encourage them to increase their MVPA but also encourage adolescents and their parents to reduce the amount of sedentary time, such as too much sitting, watching television, and other low intensity habits. When using activity monitors as a part of physical activity interventions with overweight and obese adolescents, we suggest that experts take the time to evaluate whether or not adolescents use the device. A Facebook-delivered lifestyle counselling intervention is, according to our experiences and knowledge, easy to use and it does not require expensive programmes or devices to promote physical activity among overweight and obese adolescents and reduce their sedentary time. Before the implementation process, however, more evaluation research is needed with larger numbers of overweight and obese adolescents who are sedentary at the baseline assessment. Likewise, the participant recruitment process should employ more staff to obtain larger number of participants, or perhaps a multicenter trial could be a solution.

## Figures and Tables

**Table 1:** Background characteristics of participants in the Facebook-delivered lifestyle counselling + activity monitoring group (Fb + Act), Facebook-delivered lifestyle counselling group (Fb), and control group.

	Fb + Act	Fb	Control	*p* value^a^
	(*n* = 15)	(*n* = 16)	(*n* = 15)	
Gender				
Boys (*n*) %	(5) 33	(5) 31	(4) 27	0.921
Girls (*n*) %	(10) 67	(11) 69	(11) 73	
Family				
Nuclear family (*n*) %	(9) 60	(12) 75	(11) 73	0.443
Other (*n*) %	(6) 40	(4) 25	(4) 27	

Age, years, mean (SD)	14.8 (0.8)	14.8 (0.8)	14.7 (0.8)	0.916
Weight, kg, mean (SD)	82.9 (26.6)	76.5 (15.5)	71.3 (12.8)	0.464
Height, cm, mean (SD)	166 (8.8)	166 (7.3)	162 (6.8)	0.420

BMI, mean ± (SD), kg/m^2^	29.7 (8.1)	27.5 (4.2)	27.0 (3.8)	0.904

^a^ *p* value for the differences between groups, khii^2^ for gender and family, and Kruskal-Wallis for age, weight, and height. SD: standard deviation; BMI: body mass index.

**Table 2:** Measurements at baseline (pre) and after 12 weeks (post) and changes (post-pre) in the body mass index (BMI), weight, height, and objectively measured physical activity. Mean values (standard deviation).

Measure	Fb + Act			Fb			Control		
	Pre	Post	Post-pre	Pre	Post	Post-pre	Pre	Post	Post-pre
	(*n* = 15)	(*n* = 14)	(*n* = 14)	(*n* = 16)	(*n* = 16)	(*n* = 16)	(*n* = 15)	(*n* = 14)	(*n* = 14)
BMI	29.8 (8.0)	28.5 (6.8)	−0.1 (0.9)	27.5 (4.2)	26.9 (4.2)	−0.6 (0.9)	27.0 (3.8)	26.9 (4.2)	−0.0 (0.9)
Height, cm	166 (8.8)	167 (9.7)	1.0 (0.9)	166 (7.3)	167 (7.4)	0.7 (0.6)	162 (6.8)	163 (7.3)	0.5 (1.7)
Weight, kg	83.0 (26.6)	80.7 (25.5)	0.9 (2.9)	76.5 (15.5)	75.3 (15.0)	−1.2 (2.4)	71.3 (12.8)	71.2 (13.9)	0.4 (1.8)
*Objectively measured physical activity*									
Valid measurement days									
Weekdays	13.3 (0.5)	11.8 (0.9)	−1.5 (0.4)	13.9 (0.1)	13.1 (0.6)	−0.5 (0.4)	13.3 (0.4)	12.4 (0.6)	−0.9 (0.7)
Weekend days	5.4 (0.4)	4.9 (0.5)	−0.5 (0.4)	5.8 (0.2)	5.1 (0.3)	−0.7 (0.2)	5.3 (0.3)	5.1 (0.6)	−0.2 (0.6)
Total	18.7 (0.8)	16.7 (1.3)	−1.9 (1.0)	16.7 (0.3)	18.4 (0.6)	−1.3 (0.5)	18.6 (0.7)	16.2 (1.2)	−2.4 (1.4)
Sedentary time or very light PA, min/day									
Weekdays	669.9(74.6)	560.9 (136.7)	−106.9 (114.8)	673.1 (97.2)	624.2 (105.3)	−48.9 (78.2)	704.7 (67.1)	654.4 (61.1)	−60.1 (46.2)
Weekend days	654.2 (110.7)	608.4 (108.1)	−49.4 (123.6)	656.3 (121.8)	612.4 (102.5)	−43.9 (113.6)	655.2 (71.5)	627.1 (84.6)	−37.7 (114.3)
Total	665.6 (74.7)	570.6 (119.5)	−94.5 (112.7)	668.3 (101.5)	619.5 (101.6)	−48.7 (76.9)	691.0 (66.0)	642.0 (53.5)	−58.7 (60.2)
Light PA, min/day									
Weekdays	234.0 (53.4)	274.4 (70.2)	38.0 (50.5)	251.1 (67.3)	273.3 (65.5)	22.2 (47.5)	210.0 (52.8)	240.3 (33.3)	23.4 (43.3)
Weekend days	201.3 (69.1)	256.2 (80.2)	50.7 (44.0)	223.9 (77.7)	234.4 (54.5)	10.4 (59.6)	189.1 (57.4)	232.2 (50.9)	38.9 (57.7)
Total	224.8 (55.3)	259.7 (82.3)	32.0 (51.7)	243.4 (67.0)	258.9 (57.3)	15.5 (38.6)	204.1 (50.9)	240.0 (31.9)	30.4 (38.7)
Moderate PA, min/day									
Weekdays	56.7 (20.1)	61.4 (24.6)	3.2 (20.5)	59.7 (20.1)	58.5 (23.9)	−1.2 (23.6)	48.3 (15.1)	47.6 (15.4)	−1.8 (13.7)
Weekend days	43.3 (19.1)	54.3 (16.8)	9.7 (28.6)	50.4 (24.1)	56.1 (21.3)	5.5 (18.2)	38.1 (26.4)	43.2 (15.7)	11.3 (20.0)
Total	42.4 (18.9)	57.1 (21.5)	2.8 (20.1)	56.9 (19.6)	57.6 (21.7)	0.7 (18.0)	45.4 (13.1)	45.2 (12.3)	0.7 (14.6)
Vigorous PA, min/day									
Weekdays	16.2 (10.0)	16.6 (12.0)	−0.2 (8.4)	17.4 (10.1)	16.9 (10.7)	−0.5 (9.7)	10.8 (5.8)	9.9 (5.1)	−0.7 (5.8)
Weekend days	12.3 (6.7)	16.4 (10.8)	3.6 (13.9)	15.3 (12.6)	18.9 (12.5)	3.6 (12.0)	7.3 (8.2)	9.9 (7.0)	4.5 (6.4)
Total	15.1 (8.8)	15.6 (10.3)	−0.0 (8.8)	16.8 (10.4)	17.8 (10.5)	1.0 (9.2)	9.8 (5.4)	9.7 (5.3)	0.6 (4.7)
Vigorous plus PA, min/day									
Weekdays	4.7 (5.3)	4.7 (3.9)	−0.1 (4.4)	5.5 (5.3)	5.8 (7.3)	0.3 (5.6)	2.9 (3.7)	2.1 (2.1)	−0.8 (3.9)
Weekend days	3.2 (3.4)	3.6 (3.0)	0.5 (4.8)	4.8 (5.1)	7.3 (7.0)	2.5 (6.0)	1.5 (3.1)	2.1 (2.9)	1.0 (4.1)
Total	4.3 (4.4)	4.0 (3.3)	−0.4 (3.8)	5.3 (4.9)	6.8 (7.2)	1.6 (4.4)	2.4 (3.7)	2.2 (1.9)	−0.2 (3.2)
MVPA, min/day									
Weekdays	77.7 (32.2)	82.5 (39.3)	2.7 (28.3)	82.5 (30.5)	81.8 (36.5)	−1.4 (31.9)	61.9 (20.9)	59.6 (36.5)	−3.3 (20.8)
Weekends	58.4 (26.6)	74.1 (28.8)	14.0 (44.7)	70.3 (38.9)	81.9 (36.1)	11.6 (32.7)	46.8 (35.7)	55.2 (23.7)	16.9 (26.7)
Total	72.0 (29.2)	76.8 (33.6)	2.7 (29.4)	78.9 (31.2)	82.1 (34.2)	3.1 (26.9)	57.6 (18.6)	57.3 (18.0)	1.3 (20.3)
Wearing time, min/day									
Weekdays	945.7 (69.1)	919.3 (139.9)	13.4 (90.5)	975.5 (58.8)	1020.4 (69.4)	50.5 (45.5)	974.4 (107.7)	1008.0 (80.8)	16.1 (83.2)
Weekends	914.86 (129.3)	1012.4 (90.7)	87.7 (145.1)	950.6 (70.2)	967.7 (72.2)	55.6 (71.3)	889.1 (96.2)	969.7 (94.9)	75.3 (129.6)
Total	965.7 (81.9)	983.6 (134.0)	13.0 (109.8)	960.1 (57.3)	1038.0 (68.5)	47.5 (44.3)	951.5 (96.5)	997.2 (79.0)	31.4 (94.0)
Sedentary time or very light PA as % of wearing time									
Weekdays	68 (6.4)	56 (10.6)^*∗*^	−11.9 (9.8)	67 (8.3)	59 (10.2)	−7.6 (7.5)	73 (4.4)	65 (4.7)^*∗*^	−7.2 (4.0)
Weekends	72 (8.0)	60 (8.8)	−11.3 (10.3)	69 (10.8)	61 (9.8)	−8.0 (9.7)	74 (6.3)	65 (7.0)	−9.9 (8.0)
Total	69 (6.1)	58 (10.6)	−10.4 (9.6)	67 (8.7)	60 (9.6)	−7.6 (7.1)	73 (4.2)	64 (4.1)	8.2 (4.0)

PA: physical activity, MVPA: moderate to vigorous physical activity, and ^*∗*^difference between Fb + Act and control group at posttest *p* = 0.021.

**Table 3:** Measurements at baseline (pre) and after 12 weeks (post) for self-reported physical activity and screen time. Mean values (standard deviation).

Measure	Fb + Act			Fb			Control		
	Pre	Post	Post-pre	Pre	Post	Post-pre	Pre	Post	Post-pre
	(*n* = 15)	(*n* = 14)	(*n* = 14)	(*n* = 16)	(*n* = 16)	(*n* = 16)	(*n* = 15)	(*n* = 14)	(*n* = 14)
Self-reported physical activity and screen time									
Self-reported physical activity, days out of past seven with at least 60 min MVPA	3.0 (1.8)	4.5 (1.7)	1.4 (2.9)	3.3 (1.8)	3.3 (1.8)	0.1 (1.5)	3.2 (1.3)	3.3 (1.0)	−0.1 (1.7)
Weekday screen time average hours/day									
Total	3.8 (1.9)	4.2 (2.3)	0.3 (1.8)	4.0 (2.7)	4.3 (3.9)	0.2 (1.9)	3.9 (2.2)	3.5 (1.8)	−0.5 (1.8)
Watching television, DVDs, or videos	1.4 (0.7)	1.5 (1.2)	0.7 (0.9)	1.5 (1.5)	1.4 (1.2)	−0.1 (0.9)	1.4 (1.0)	1.4 (0.9)	−0.1 (0.8)
Playing computer or video/console games	0.8 (1.0)	0.8 (1.0)	−0.1 (0.7)	1.0 (1.2)	1.3 (1.7)	0.3 (1.4)	0.7 (1.0)	0.5 (0.8)	−0.1 (1.4)
Computer use for reasons other than playing	1.5 (1.2)	2.0 (1.3)	0.4 (1.2)	1.6 (1.0)	1.7 (1.3)	0.1 (0.9)	1.8 (1.4)	1.6 (1.5)	−0.2 (0.9)
Weekend screen time average hours/day									
Total	6.1 (2.2)	5.7 (2.8)	−0.4 (1.1)	6.3 (3.3)	6.0 (3.9)	−0.3 (2.4)	5.6 (2.1)	4.8 (2.2)	−0.7 (1.9)
Watching television, DVDs, or videos	2.4 (1.1)	1.9 (1.2)	−0.6 (1.1)	2.4 (1.8)	1.9 (1.3)	−0.5 (1.0)	1.8 (1.3)	1.9 (1.2)	−0.0 (1.0)
Playing computer or video/console games	1.5 (1.6)	1.5 (1.4)	−0.1 (1.4)	1.8 (1.8)	1.8 (1.9)	−0.1 (1.8)	1.1 (1.1)	0.7 (1.1)	−0.4 (1.3)
Computer use for reasons other than playing	2.3 (1.5)	2.3 (1.5)	−0.1 (1.8)	2.0 (1.4)	2.3 (1.4)	0.3 (1.5)	2.5 (1.5)	2.3 (1.5)	−0.3 (0.8)

PA: physical activity; MVPA: moderate to vigorous physical activity.
